# Clinical and histopathological differential diagnosis of Laugier‐Hunziker syndrome: An extremely rare case with unusual extensive oral hyperpigmentation

**DOI:** 10.1002/ccr3.3522

**Published:** 2020-11-16

**Authors:** Verena Toedtling, Fiona Carol Crawford

**Affiliations:** ^1^ Division of Dentistry School of Medical Sciences Faculty of Biology, Medicine and Health, Oral and Maxillofacial Surgery The University of Manchester Manchester UK; ^2^ Retired Oral Medicine Consultant Edinburgh UK

**Keywords:** hyperpigmentation, Laugier‐Hunziker syndrome, mucocutaneous pigmentary disorder, Peutz‐Jegher syndrome, physiological melanoplakia

## Abstract

Laugier‐Hunziker syndrome is a rare and benign disorder characterized by hyperpigmentation of the lips and buccal mucosae with associated longitudinal melanonychia of nails. Clinical correlation is needed to rule out other pigmentary disorders.

## Graphical abstract

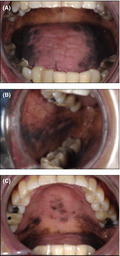

## 1 INTRODUCTION

Laugier‐Hunziker syndrome, an extremely rare and benign idiopathic disorder, characterized by asymptomatic diffused macular hyperpigmentation of the lips/buccal mucosae with longitudinal melanonychia of the nails. We present a case of a 46‐year‐old South Asian woman with characteristic clinical‐histopathological features and unusual extensive hyperpigmentation of the lateral borders of tongue and mixed connective tissue disease.

Laugier‐Hunziker syndrome (LHS) was first described in 1970 as an extremely rare and benign idiopathic disorder characterized by asymptomatic macular hyperpigmentation of the lips and buccal mucosae with associated longitudinal melanonychia of the nails.^1^ Given the large number of cases of oral pigmentation, it was suggested that LHS could only be confidently diagnosed in the presence of both oral and ungual pigmentation and the absence of underlying systemic disease.^2^ Due to the original observation, only around 172 cases have been described, including cases that have been reported in related family member and very few other cases with nonclassical features or atypical oral and cutaneous presentations.^3^, ^4^

## 2 CASE HISTORY

We present a case in a 46‐year‐old South Asian female with characteristic clinico‐histopathological features of LHS. The patient's main concern was the increasingly visible black pigmentation of her lower lip and tongue which she developed gradually over 10 years. There were no other pigmentations elsewhere on clinical examination. She presented with a medical history of interstitial lung disease in association with mixed connective tissue disorder. Her BMI, blood pressure, fasting blood sugar test, FBC, plasma cortisol, and adrenocorticotrophic hormones (ACTH) were normal, and she had a negative heavy metal exposure and malnutrition history. Her previous endoscopy was normal and showed no evidence of polyposis. In addition to this, she is a professional teacher, did not appear malnourished on clinical presentation, and reported to be a nonsmoker. There was no family history of hyperpigmentation, skin disease or gastrointestinal disorders, and no relevant drug intake prior to the onset of the pigmentation.

**FIGURE 1:**
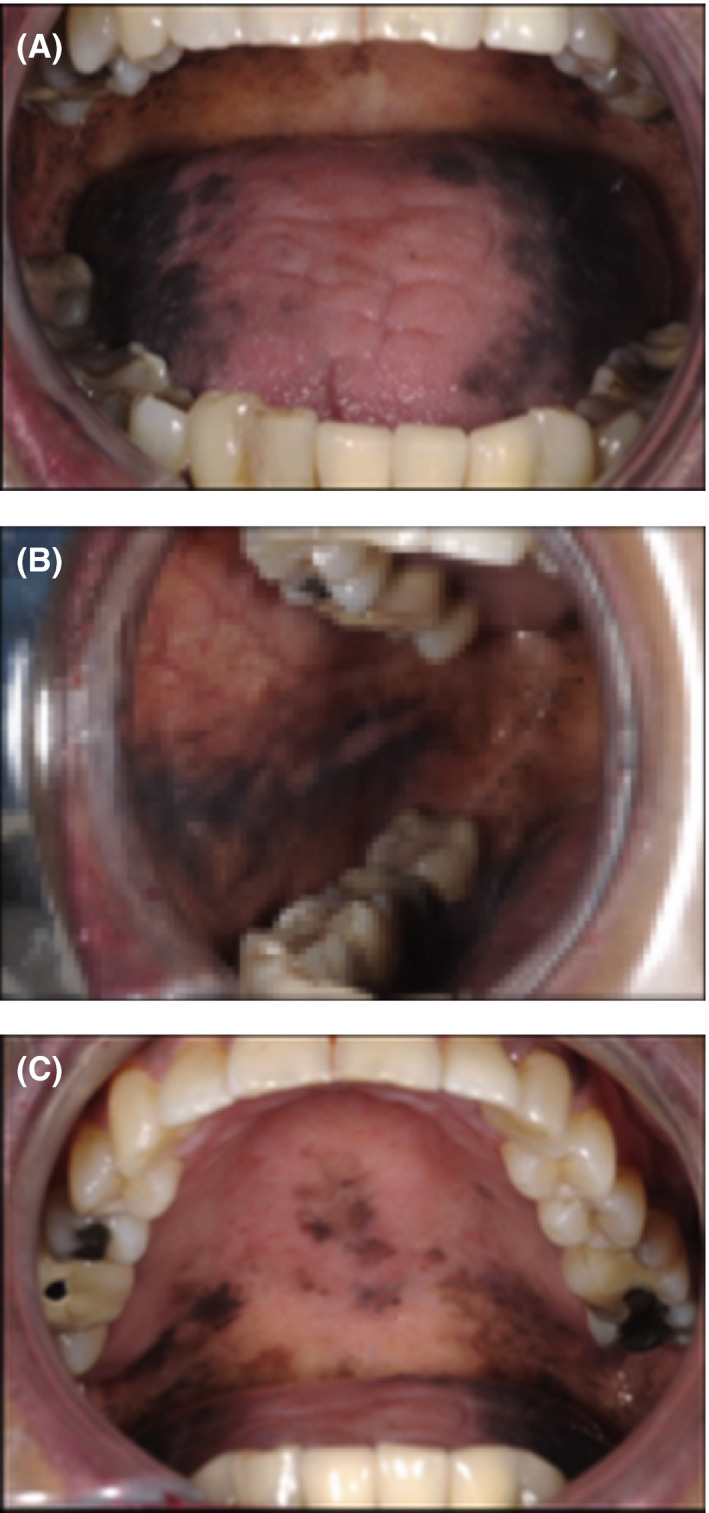
A, Lateral boarders of the tongue. B, Buccal mucosa. C, Palate

**FIGURE 2:**
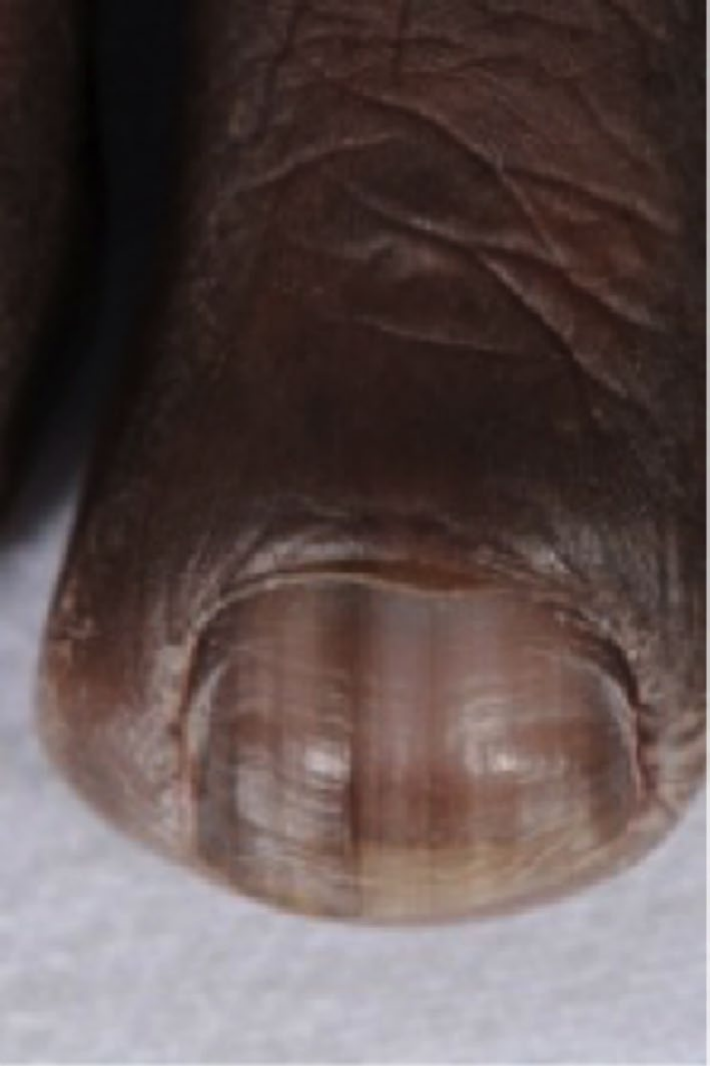
Toenail with one longitudinal band and pigmentation invoving the lateral aspects of the nail plate and proximal nail fold (pseudo‐Hutchinson's sign)

On examination, there was evidence of a confluent lenticular hyperpigmentation of the lateral borders of the tongue, lower lip, buccal mucosae, gingivae, and hard and soft palate (Figure 1A,B,C). Pigmented striae on multiple fingernails and toenails were reported by the patient, and once varnish and acrylic nails were removed from two toenails, longitudinal melanonychia became evident (Figure 2).

**FIGURE 3:**
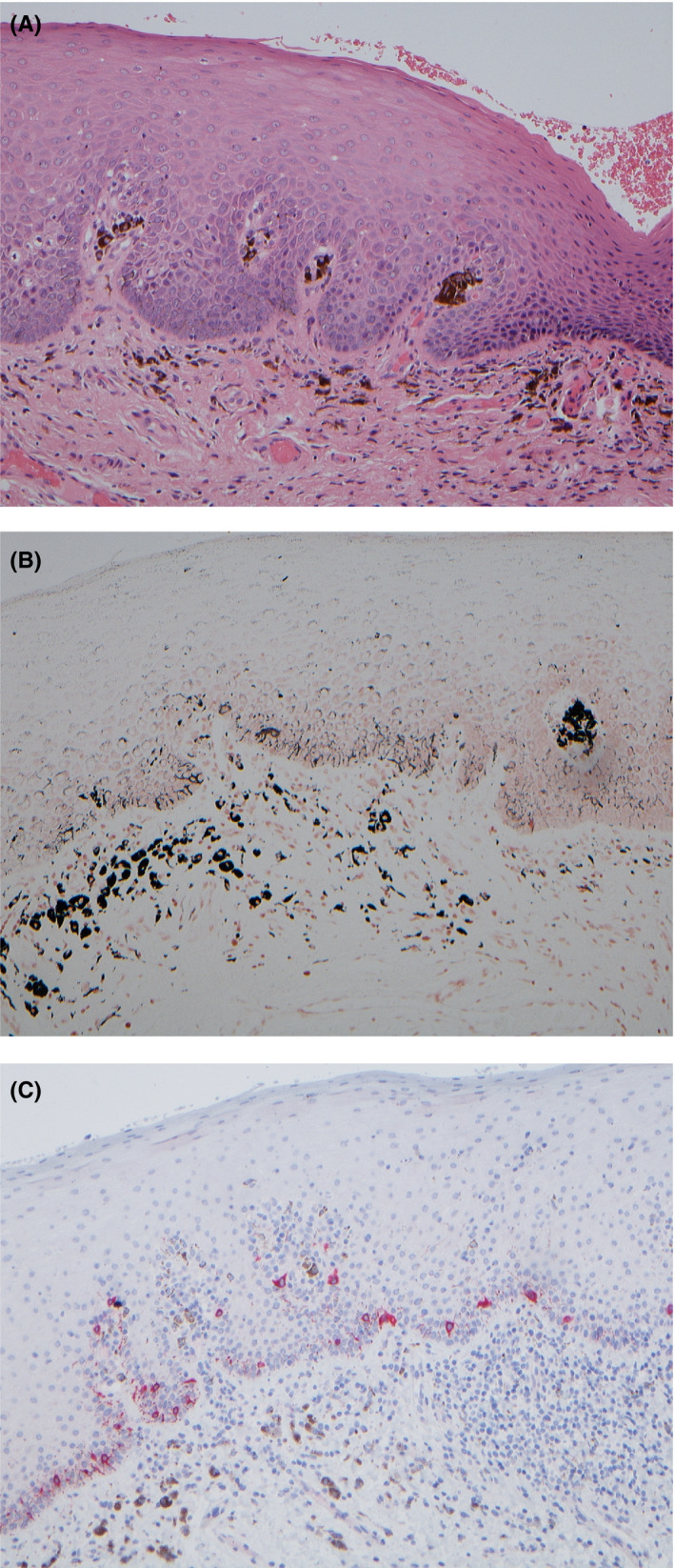
A, Parakeratosis and acanthosis of the stratified squamous epithelium and clusters of melanophages within the connective tissue papilla, H&amp;E ×100. B, Masson‐Fontana silver stain which stains melanin pigment black thus proving the brown pigment is melanin (Perls stain for hemosiderin was also performed and was negative), ×100. C, S100 Immunohistochemistry performed with red counterstain and shows the melanocytes in the basal layer of the epithelium. They are normal in number and distribution. Visible is also the contrast between the red staining, melanocytes, and the brown melanophages in the connective tissue, ×100

The histological examination of the incisional biopsy which was taken from the buccal mucosa showed that melanin was confined to the basal layer and was phagocytosed within macrophages in the upper connective tissue. There were foci of lymphocytic infiltrate in the basal layer associated with spongiosis and occasional cytoid bodies. There was no evidence of dysplasia or malignancy (Figure 3A,B,C). The clinical and histological findings were correlated, and the diagnosis of Laugier‐Hunziker syndrome was made and excluded melanoma from the differential diagnosis.

## 3 DISCUSSION

In 1970, Laugier and Hunziker reported five cases of acquired macular hyperpigmentation of the oral mucosa membranes and two cases also displayed pigmentary changes of the nails. Generally, the color of the oral lesions ranges from slate to brown‐black. They may be solitary or confluent round, lenticular, and linear lesions with variable sizes that range from 5 mm to 1 cm in diameter. Most commonly affected are the lower lip, buccal mucosa, and palate. The tongue, floor of the mouth, and gingiva are rarely involved.^1^, ^5^

Our reported case showed unusually marked lingual hyperpigmentation which has previously not been widely reported in the literature but supports Litoyo and Miyazawa's observations in 1999. They followed up a LHS case for 8 years and observed a sharp increase in the pigmented maculae each year, with some of the maculae darkening and fusing together.^6^

In cases with LHS, oral pigmentation may exist alone or in combination with longitudinal nail striae. 60% of patients have nail involvement, and fingernails are more frequently affected then toenails. Characteristically one or more longitudinal bands of pigment are present which can vary in intensity and width but arise without nail dystrophy.^7^, ^8^, ^9^ At times, the melanonychia is accompanied by pigmentary changes of the proximal nail fold, termed the Hutchinson's or pseudo‐Hutchinson's sign which was also observed in our case. Interestingly, the literature showed that this has a benign progression when occurring in association with LHS.^9^

Extended mucocutaneous pigmentations are rare but have become a documented feature of LHS. There have been reports of the neck, esophagus, thorax, dorsal/lateral aspect of fingers, palms, soles, the genitalia, sclera, and conjunctiva being affected.^4^, ^10^, ^11^, ^12^ The etiology of the abnormal melanin production is unknown, and no environmental risk factors have been identified.^13^ To date, we are only aware of one familial case has been described by Makhoul in 2003 involving a mother and two daughters.^1^, ^14^

The histopathology of this case is entirely in keeping with the past ultrastructural studies which have demonstrated that LHS generally shows an increased melanocytic activity with accumulation of melanophages but no increase in the number of melanocytes.^15^, ^16^, ^17^ No consistent systemic association with LHS has so far been recognized; however, some cases have been reported in patients with Sjogren's syndrome, lupus erythematosus, auto‐immune hemolytic anemia, and inflammatory arthritis. Interestingly, our case was also known to have a mixed connective tissue disease and a causal association with LHS has previously been discussed and hypothesized in the literature.^1^ LHS predominates in adults with a mean age of 52 and median age of 42 years. It has previously been reported more frequently in females but is now thought to have an equal distribution between sexes. Only very few case have been reported in the English literature, mostly in dermatological journals.^1^, ^2^ LHS is mainly documented in whites,^18^ and the prevalence appears to be higher in Western Europeans^1^ but has also been described in people of Asian, Arabic, Hispanics, and in our reported case Pakistani descent.^19^, ^20^, ^21^

Racial and ethnic melanosis is most commonly noted on the gingivae, and Fry and Almeyda in 1968 found that idiopathic buccal melanosis without longitudinal melanonychia is a normal finding in 38% of Negroids and 5% of Caucasoid. It characteristically occurs during the first to third decade. Melanonychia striata without associated mucosal melanosis is a normal finding in 77% of black patients in the second decade and 90% of blacks when they are aged 50 or over but generally this is confined to the thumbnail. The prevalence of oral and ungula hyperpigmentation in other dark‐skinned races is not precisely known.^22^

Peutz‐Jegher syndrome (PJS) is an important differential diagnosis of LHS. It can manifest itself with small maculae on and around the lips which are usually present at birth. The cutaneous hyperpigmentation usually fades with puberty; however, oral lesions persist. PJS follows an autosomal dominant inheritance pattern and is associated with hamartomatous polyposis. Carriers have an increase risk of developing recurrent bowel obstructions, gastrointestinal bleeding, and a higher risk to malignancy justifying intensive screening protocols. Reports have shown that as high as 48% of affected people usually die of malignancy before the age of 60 years. Longitudinal pigmented bands on nails have been reported in association with this syndrome but are extremely rare, and none of the cases reported showed bowel abnormalities.^23^, ^24^

We believe that the incidence of LHS has been under reported and that at least some of the cases were diagnosed with physiological melanoplakia or PJS which showed attenuated manifestations. Nonetheless, there are some distinct characteristics (Table 1) which may help to differentiate between physiological melanoplakia and the two syndromes.

**TABLE 1:** Differential diagnoses

	Physiological melanoplakia	Laugier‐Hunziker syndrome	Peutz‐Jegher syndrome
Genetic background	Present	No genetic factors^25^	Autosomal dominant
Onset of oral pigmentation	Infancy/Puberty	40s‐50s	Birth/Infancy
Gastrointestinal polyposis	Absent	Absent	Present
Location of frequent oral pigmentation	Gingiva Buccal mucosa	Lips Buccal mucosa	Lips Perioral
Nail pigmentation	77% of Negroids	60%	Extremely rare

## 4 CONCLUSION

The importance of LHS is related to it being included in the differential diagnosis of pigmentation of the oral mucosa and nail apparatus, occurring in middle‐aged patients including dark‐skinned races. At present, systemic illness and malignancy are not recognized features of LHS, and it is therefore important to rule out other mucocutaneous pigmentary disorders that do require screening and medical intervention, such as PJS. Therefore, precise and prompt clinical recognition of LHS allows early reassurance to patients and reduces the need for invasive and often also regular as well as expensive procedures and treatments.

### 4.1 Additional relevance

The case is presented with regard to the unusual marked bilateral lingual hyperpigmentation which has previously not been widely reported in the literature and its comorbid connective tissue disease.

## CONFLICT OF INTEREST

The authors declare no conflict of interests.

## AUTHOR CONTRIBUTION

Dr VT and Dr FC: has made substantial contribution to conception and design, acquisition of data, and analysis and interpretation of data, has been involved in drafting the manuscript, revising it critically for important intellectual content, and has given final approval of the revision to be published.

## ETHICAL APPROVAL

Informed consent was obtained from the patient for publication of information.

## Data Availability

The data that support the findings of this study are available on request from the corresponding author. The data are not publicly available due to privacy or ethical restrictions.
